# cfDNA correlates with endothelial damage after cardiac surgery with prolonged cardiopulmonary bypass and amplifies NETosis in an intracellular TLR9-independent manner

**DOI:** 10.1038/s41598-017-17561-1

**Published:** 2017-12-12

**Authors:** Adnana Paunel-Görgülü, Max Wacker, Mouhamed El Aita, Shoreshfan Hassan, Georg Schlachtenberger, Antje Deppe, Yeong-Hoon Choi, Elmar Kuhn, Thorsten O. Mehler, Thorsten Wahlers

**Affiliations:** 1 Department of Cardiothoracic Surgery, Heart Center of the University of Cologne, Cologne, Germany; 2 Department of Anaesthesiology and Intensive Care Medicine, University Hospital Cologne, Cologne, Germany

## Abstract

Cardiopulmonary bypass (CPB) provokes inflammation culminating in organ dysfunction and increased mortality. Recently, neutrophil extracellular traps (NETs) have been found to be involved in a variety of cardiovascular diseases promoting tissue and organ injury. Here, we aimed to elaborate the proinflammatory potential of circulating cell-free (cf)DNA in patients undergoing cardiac surgery with CPB. Plasma was collected pre- and postoperatively as well as at d1, d3, d5 and d8 after surgery. At d1, we found circulating cfDNA levels to be significantly increased in patients with prolonged CPB duration (>100 min) when compared to those with shorter CPB times (CPB < 100 min). Increased CPB duration yielded in higher levels of circulating mitochondrial (mt)DNA, soluble thrombomodulin (sCD141) and ICAM-1, reflecting endothelial damage. Positive correlation between cfDNA and sCD141 was demonstrated at all time points. Plasma and cfDNA from patients with CPB > 100 min induced NETs release by neutrophils from healthy donors which was not suppressed by inhibitors of intracellular toll-like receptor (TLR)9. DNA binding to neutrophils’ surface (s)TLR9 has been evidenced. Altogether, we demonstrate that elevated plasma cfDNA might be useful to assess CPB-mediated detrimental effects, including endothelial damage, in cardiac surgical patients with prolonged CPB duration. cfDNA-triggered NETosis is independent of classical TLR9 signaling.

## Introduction

Cardiac surgery with cardiopulmonary bypass (CPB) support initiates a systemic inflammatory response (SIRS), presumably caused by contact of blood components with the artificial surface of the extracorporeal circuit, that is associated with postoperative morbidity and mortality^1^. In this regard, many studies demonstrated increased inflammatory markers, such as TNF-α, IL-6, IL-8 after cardiac surgery with CPB^2,3^. Massive activation of leukocytes, e.g. neutrophils, and different biochemical pathways may result in microthrombosis, microemboli and depletion of coagulation factors. Neutrophil-derived enzymes, such as elastase and myeloperoxidase (MPO) and reactive oxygen species (ROS) contribute to tissue injury and endothelial dysfunction, predisposing patients to organ injury. Further on, activated neutrophils also directly activate endothelial cells thereby increasing perivascular edema and leukocyte transmigration into extracellular matrix^4^. Recently, the release of neutrophil extracellular traps (NETs)/cell-free DNA (cfDNA), by a process termed NETosis, and their potent proinflammatory and cytotoxic effects have gained much attention as risk factors for cardiovascular diseases as well as the development of postoperative complications^5–7^. NETs are web-like structures composed of decondensed chromatin and antimicrobial proteins that can entrap pathogens but also contribute to the pathophysiology of multiple inflammatory diseases such as myocardial ischemia/reperfusion injury and stroke^7,8^. Many physiological inducers of NETosis have been reported, including microorganisms^9^, activated platelets^10^, activated endothelial cells^11^ and proinflammatory cytokines^12^. However, inappropriate NETs release may cause tissue damage and inflammation. Previous studies have shown, that MPO and histones are responsible for NETs-mediated endothelial and epithelial cell cytotoxicity^13^. Additionally, NETs ingredients might degrade inhibitors of coagulation favoring intravascular thrombus formation^14^. Notably, marked increase in NETs formation in patients undergoing elective cardiac surgery and correlation with perioperative renal dysfunction was reported^15^. However, NETosis does not mandatory require neutrophil death and few years ago NETs release by viable neutrophils has been demonstrated, whereby these structures are formed from pure mitochondrial DNA (mtDNA)^16^. In addition, release of nuclear DNA and mtDNA upon neutrophil stimulation with PMA and NO has also been demonstrated^17^.

Human mitochondrial DNA (mtDNA) consists of an approximately 16.5 kb circular, double-stranded extrachromosomal DNA and might contain high amounts of unmethylathed CpG. Recent research has implicated mtDNA as a damage-associated molecular pattern (DAMP) and marked increase in extracellular mtDNA was already found in different pathological disorders, e.g after cardiac surgery^18^ and during sterile SIRS^19^. mtDNA fragments participate in different kinds of innate immune modulation by activating pattern recognition receptors, of which toll-like receptors (TLRs) are the most prominent one. Proinflammatory mtDNA mediates inflammatory responses through CpG/TLR9 interactions, supporting neutrophil activation and TLR9 inhibition significantly attenuates mtDNA-induced systemic inflammation in mice^20^. Recently, a study based on multiple cohorts showed that mtDNA can improve risk prediction and there is a tight relationship between elevated plasma mtDNA level and 28-day mortality^21^. Postoperative inflammatory responses are highly related to the prognosis of cardiac surgery. However, the impact of CPB on neutrophil TLR9 expression and circulating cfDNA as well as the potential relevance of cfDNA for patients’ outcome has not been reported until now. Here, we hypothesize that circulating cfDNA might reflect the onset of CPB-induced systemic inflammation in patients undergoing cardiac surgery. We further sought to evaluate how cfDNA might amplify neutrophil-mediated inflammatory reactions and to further elucidate the significance of the classical DNA receptor TLR9 in this process.

## Results

### Patient demographics and clinical scores

Patients’ baseline demographics, surgery information as well as physiologic parameters are summarized in Table 1. Among all patients twenty-two underwent cardiac surgery with CPB < 100 min and twenty-six patients underwent cardiac surgery with CPB > 100 min. Mean age at the time of investigation did not differ between the two groups. Both groups had comparable cardiovascular comorbidities such as hypertension, COPD and diabetes mellitus. Most of the patients included in the CPB < 100 min group underwent aortic valve replacement, whereas aortic valve replacement combined with coronary artery bypass grafting represented the most performed surgical procedure on patients with CPB > 100 min. Significant increase in aortic cross-clamping time, days of hospitalization and EuroSCOREs was found in patients with CPB > 100 min. Among the study population one patient died postoperatively at day 7.

**Table 1:** Baseline characteristics and surgical procedures.

	Short-CPB (<100 min)	Long-CPB (>100 min)	*P* value
N	22	26	
Age (years)	64.88 ± 13	68.15 ± 12.3	0.45
Female	11(50%)	8(30.7%)	0.183
BMI	28.6 ± 5.8	27.5 ± 5.0	0.68
Aortic cross-clamping time (min)	42.7 ± 21.4	87.4 ± 24.1	**<0.0001**
CPB (min)	80.7 ± 13.6	148.2 ± 71.3	**<0.0001**
In-hospital mortality (%)	4, 3	0	
ICU stay (d)	2.9 ± 2.9	3.4 ± 4.1	0.68
Hospital stay (d)	10.0 ± 6.3	11.8 ± 5.4	**0.047**
Ventilation time (h)	16.41 ± 6.2	40.9 ± 85.5	0.19
Euroscore additive	5.5 ± 3.2	7.5 ± 3.3	**0.038**
Euroscore logistic	7.2 ± 6.7	12.4 ± 9.9	**0.039**
SAPS II	28.5 ± 8.9	29.8 ± 7.4	0.71
TISS	28.3 ± 3.4	28.9 ± 3.7	0.98
Risk factors			
Diabetes mellitus	5(31.8%)	7(30%)	0.67
COPD	3(13.64%)	4(15.4%)	0.77
Hypertension	14(68.2%)	21(80.8%)	0.43
Operative procedure			
CABG	4	3	
AV replacement	14	7	
CABG + AV replacement	1	9	
MV replacement	0	1	
MV repair	0	1	
MV repair + TV replacement	0	1	
CABG + MV repair	0	1	
LVAD	2	0	
ASD closure	1	0	
Aortic surgery + AV replacement	0	1	
Aortic surgery + AV + MV replacement	0	1	
Aortic surgery + CABG	0	1	

***ASD*** atrial septal defect; *BMI* body mass index; *CPB* cardiopulmonary bypass; *ICU* intensive care unit; *COPD* chronic obstructive pulmonary disease; *CABG* coronary artery bypass grafting; *AV* aortic valve; *MV* mitral valve; *LVAD* left ventricular assist device; ***TV*** tricuspid valve replacement. Data are presented as mean ± SD.

Table 2 summarizes the biochemical and clinical parameters of patients after on-pump surgery. Characteristics of patients undergoing off-pump artery bypass grafting (OPCAB) without CPB can be found as Supplementary Table S1.

**Table 2:** Clinical data.

	Short-CPB (CPB < 100 min)	Long-CPB (CPB > 100 min)	*P* value
N	22	26	
Noradrenaline (hours)	20.45 ± 17.6	23.69 ± 21.15	0.57
Dobutamine (hours)	7.68 ± 7.19	11.31 ± 15.71	**0.0006**
Corotrope (hours)	0.31 ± 1,57	0.36 ± 1.71	0.91
Rethorakotomie (%)	4.55	3.85	0.91
Antibiotic (%)	22.73	30.77	0.54
Pneumonia (%)	4.55	7.69	0.26
Wound infection (%)	9.09	7.69	0.86
Delirium (%)	0	15.38	**0.0001**
PDT (%)	0	3.85	**0.0001**
Creatinine preoperative (mg/dl)	0.97 ± 0.34	1.10 ± 0.32	0.185
Creatinine (max.)	1.23 ± 0.87	1.34 ± 0.67	0.61
Acute kidney failure (%)	9.09	15.38	0.52
CRP (max.)	201.62 ± 85.36	197.47 ± 68.23	0.85
CK (max.)	921.14 ± 757.03	1041.1 ± 336.4	0.054
CK-MB (max.)	44.18 ± 27.31	55.19 ± 22.6	**0.048**
Postoperative infarct (%) based on CK	54.55	65.38	0.98

ICU intensive care unit; PDT percutaneous dilatation tracheotomy defect; CRP C-reactive protein; CK Creatinine Kinase, CK-MB Creatine Kinase Muscle and Brain. Data are presented as mean ± SD.

### Elevated levels of circulating cfDNA and altered neutrophil TLR9 expression in patients with long-term CPB

**Figure 1:**
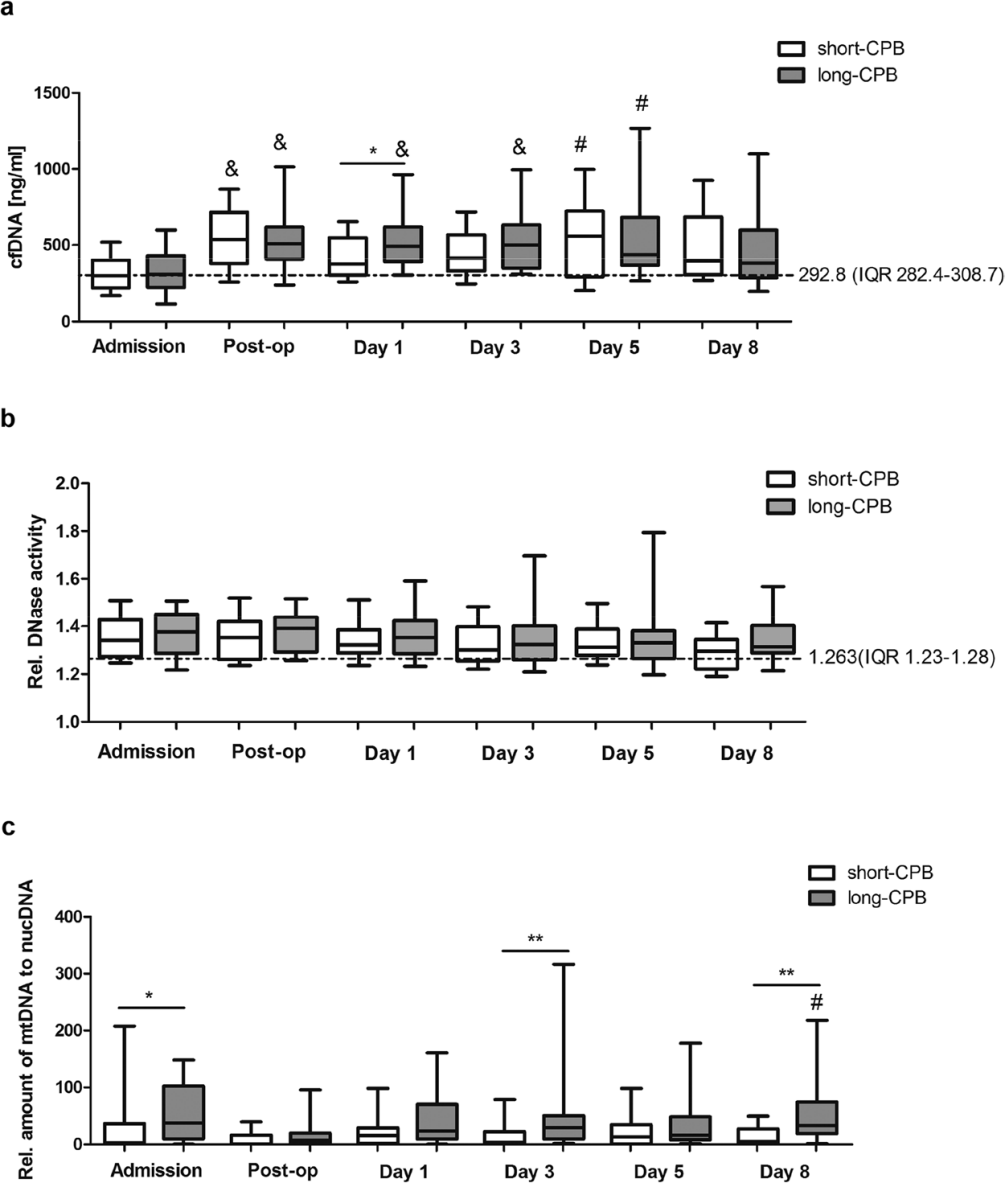
Kinetics of cfDNA and plasma *DNase I* activity in patients undergoing cardiac surgery with CPB. (**a**) Plasma levels of cfDNA were determined in cardiac surgery patients with CPB < 100 min (short-CPB; Admission n = 22; Post-op n = 19; d1 n = 20; d3 n = 19; d5 n = 17; d8 n = 13) and CPB > 100 min (long-CPB; Admission n = 26; Post-op n = 19; d1 n = 24; d3 n = 25; d5 n = 25; d8 n = 21) before and after surgery at defined times. The dotted line indicates levels quantified in plasma of eight healthy volunteers (median with IQR (interquartile range)). ^#^p < 0.05; ^&^p < 0.01 vs. admission; *p < 0.05. (**b**) Relative plasma *DNase I* activity was quantified in patients with CPB < 100 min (Admission n = 21; Post-op n = 17; d1 n = 19; d3 n = 19; d5 n = 18; d8 n = 13) and CPB > 100 min (Admission n = 26; Post-op n = 17; d1 n = 20; d3 n = 24; d5 n = 23; d8 n = 19) as described in Methods. Five healthy volunteers served as controls (dotted line; median with IQR). (**c**) Circulating cfDNA was isolated from patients’ plasma and the content of mitochondrial DNA (mtDNA) and nuclear DNA (nucDNA) was determined by Real time PCR using gene specific primers. Relative amount of mtDNA to nucDNA in both, patients with CPB < 100 min (Admission n = 18; Post-op n = 17; d1 n = 20; d3 n = 19; d5 n = 17; d8 n = 13) and CPB > 100 min (Admission n = 20; Post-op n = 16; d1 n = 17; d3 n = 21; d5 n = 21; d8 n = 18), is depicted. ^#^p < 0.05 vs. Post-op; *p < 0.05; **p < 0.01.

In order to study the impact of CPB duration on the onset of inflammation, we first quantified levels of cfDNA in patients’ plasma at the time of admission and postoperatively at defined time points. Plasma cfDNA levels strongly increased after surgery in patients undergoing cardiac surgery with CPB < 100 min (short-CPB) and CPB > 100 min (long-CPB), suggesting activation of proinflammatory pathways by the extracorporeal circuit (Fig. 1a). In patients with CPB > 100 min, cfDNA levels remained significantly elevated at least until day 5 and significant intergroup differences were already detected at day 1 after surgery. No significant increase of cfDNA was observed in patients undergoing OPCAB surgery (Supplementary Fig. S1), indicating that cfDNA increase doesn’t represent a common inflammatory response induced by the surgical trauma. Importantly, increase in circulating cfDNA was not related to a reduced plasma *DNase I* activity as no alternations in *DNase I* activity were detected over time (Fig. 1b). Thus, elevated cfDNA levels are attributed to an increased number of NETing neutrophils or damaged cells, respectively.

**Figure 2:**
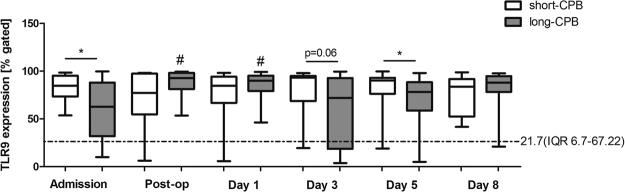
Effect of CPB duration on neutrophil TLR9 expression. Neutrophils were isolated from patients with CPB < 100 min (short-CPB; Admission n = 21; Post-op n = 11; d1 n = 20; d3 n = 21; d5 n = 18; d8 n = 13), and CPB > 100 min (long-CPB; Admission n = 26; Post-op n = 13; d1 n = 24; d3 n = 23; d5 n = 23; d8 n = 21) at different times before and after surgery and total TLR9 protein expression was analyzed in permeabilized cells by flow cytometry. Percentage of TLR9 expressing cells is depicted. The dotted line indicates TLR9 expression (median with IQR) in neutrophils of eight healthy volunteers. ^#^p < 0.05 vs. admission; *p < 0.05.

Recent work has demonstrated that mitochondrial DNA (mtDNA) might represent an ingredient of NETs structures among others^17^ and its proinflammatory property in DAMP-associated inflammation is well documented^20^. We therefore questioned if mtDNA might also be detected in circulating cfDNA in patients’ plasma. Real time PCR analyses of DNA isolated from plasma samples revealed that mtDNA levels were significantly increased in patients with long-CPB vs. those with short-CPB at admission, indicating that patients included in the long-CPB group display a more severe disease and are more prone for systemic inflammation (Fig. 1c). Circulating mtDNA levels markedly increased postoperatively in patients with long-term CPB, especially at day 3 and day 8 after surgery. At these times, significant intergroup differences were observed. mtDNA is known to activate neutrophils through TLR9/p38 MAPK^22^ and it has already been reported that it might upregulate TLR9 expression^23^. Here, patients undergoing cardiac surgery with CPB > 100 min displayed significantly reduced neutrophil TLR9 baseline levels at admission vs. patients with short-CPB, although TLR9 levels were found to become significantly upregulated immediately after surgery as well as at day 1 (Fig. 2). However, neutrophil TLR9 levels remained clearly reduced at day 3 and day 5 after surgery when compared to patients with shorter CPB duration, beside higher levels of circulating mtDNA. Compared to healthy controls (median 21.7, IQR 6.7–67.22), all patients had significantly elevated neutrophil TLR9 levels at admission time (**p < 0.01 vs. short-CPB (median 84.64; IQR 73.4–95.33); *p < 0.05 vs. long-CPB (median 62.63; IQR 31.9–88.1)).

### Positive correlation between cfDNA and endothelial damage in patients with long CPB duration

**Figure 3:**
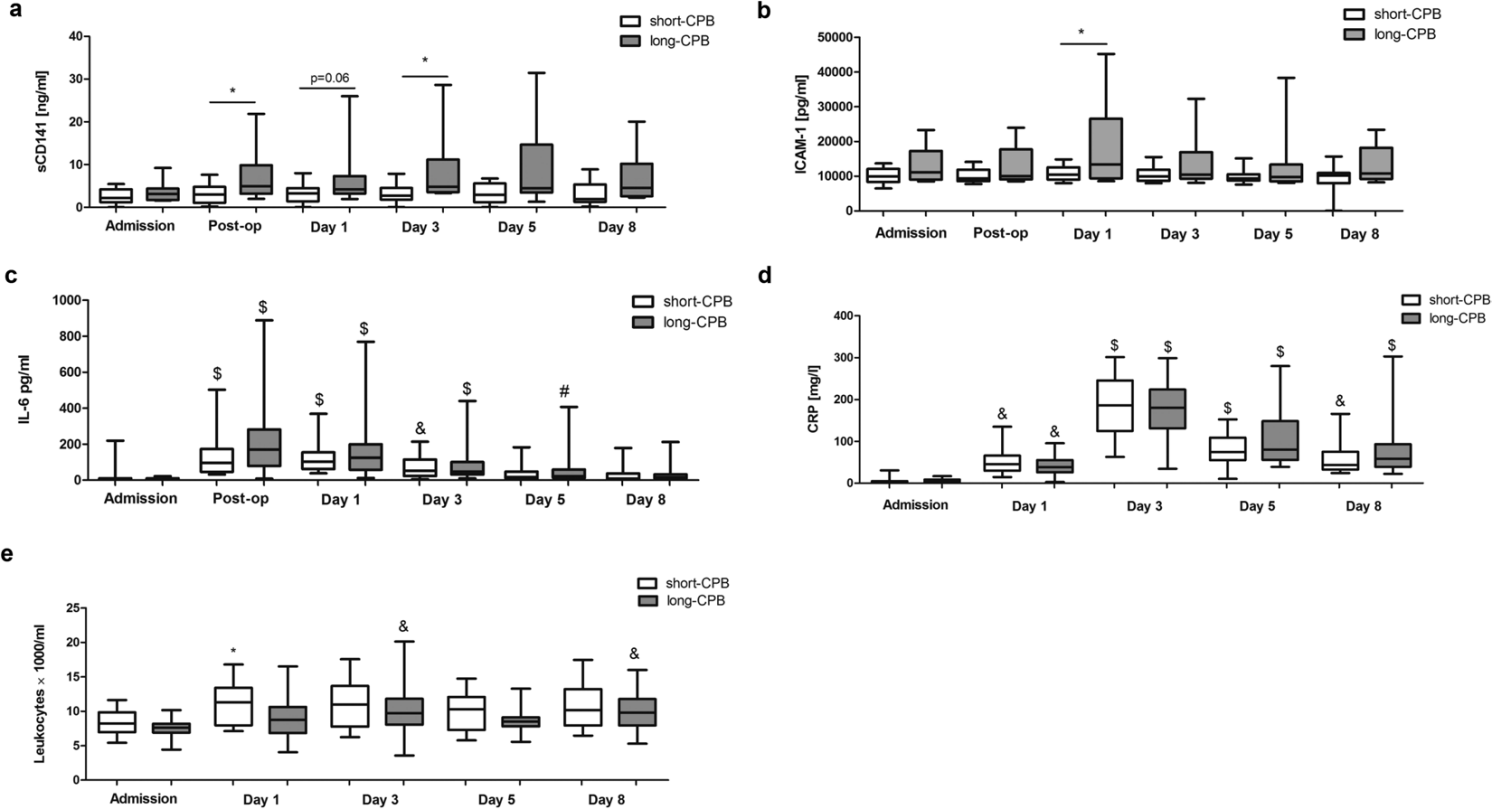
Plasma levels of inflammatory biomarkers and leukocyte counts in patients undergoing cardiac surgery with CPB. Levels of soluble thrombomodulin (sCD141, (**a**) short-CPB (Admission n = 20; Post-op n = 18; d1 n = 20; d3 n = 19; d5 n = 17; d8 n = 13); long-CPB (Admission n = 22; Post-op n = 14; d1 n = 20; d3 n = 19; d5 n = 21; d8 n = 15)), ICAM-1 (**b)** short-CPB (Admission n = 15; Post-op n = 12; d1 n = 13; d3 n = 15; d5 n = 14; d8 n = 11); long-CPB (Admission n = 14; Post-op n = 11; d1 n = 13; d3 n = 16; d5 n = 16; d8 n = 15)) and IL-6 (**c**) short-CPB (Admission n = 19; Post-op n = 18; d1 n = 20; d3 n = 21; d5 n = 20; d8 n = 14); long-CPB (Admission n = 20; Post-op n = 16; d1 n = 21; d3 n = 21; d5 n = 22; d8 n = 20)) were quantified in patients’ plasma by commercially available Elisas at the times indicated. CRP levels (**d)** short-CPB (Admission n = 22; d1 n = 22; d3 n = 20; d5 n = 20; d8 n = 16); long-CPB (Admission n = 26; d1 n = 26; d3 n = 26; d5 n = 26; d8 n = 25)) and leukocyte counts (**e**) short-CPB (Admission n = 22; d1 n = 22; d3 n = 22; d5 n = 21; d8 n = 18); long-CPB (Admission n = 26; d1 n = 26; d3 n = 26; d5 n = 26; d8 n = 25)) were measured routinely in all patients. *p < 0.05; ^&^p < 0.01, ^$^p < 0.001 vs. admission.

**Figure 4:**
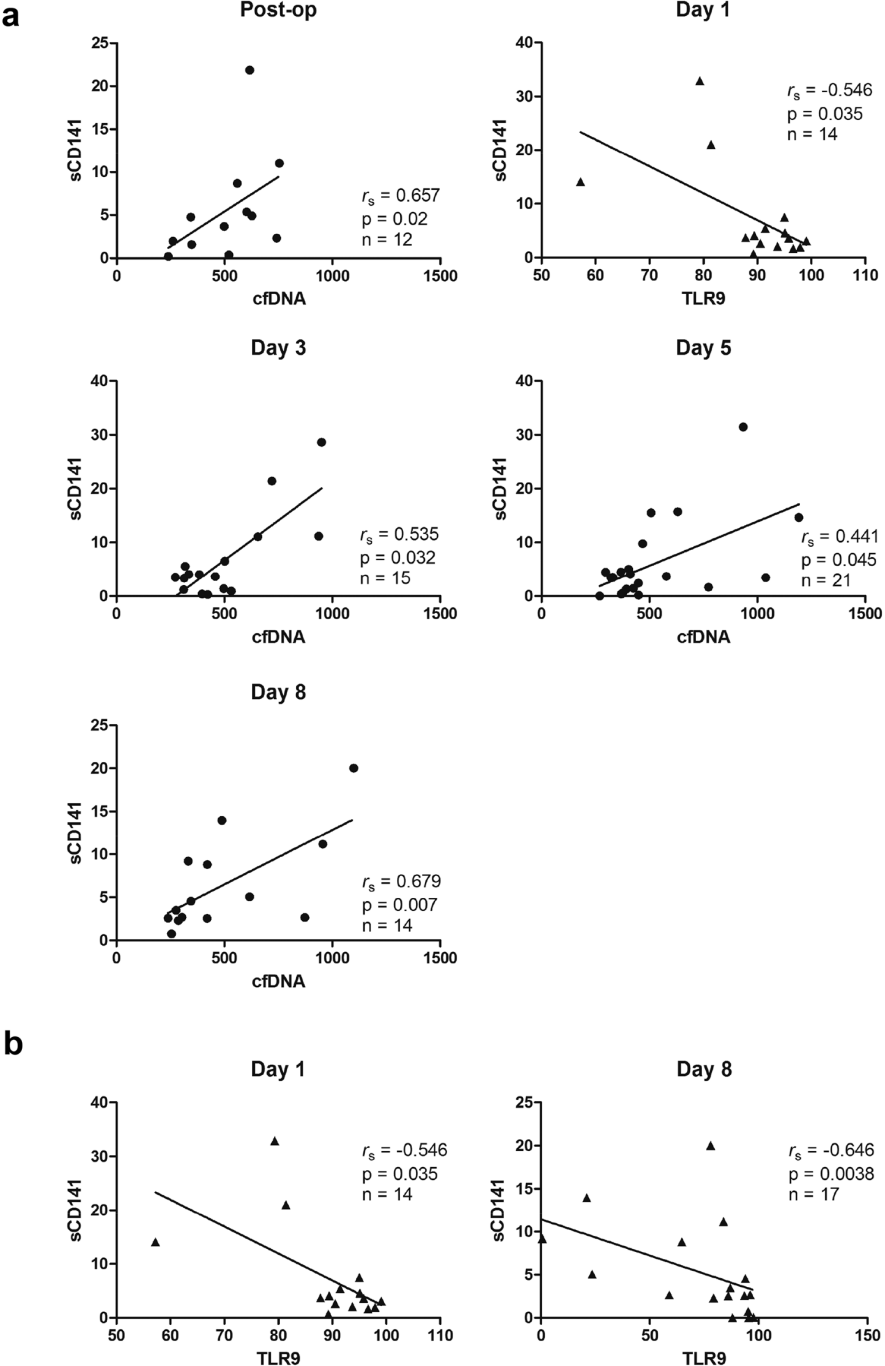
Correlation between sCD141 and neutrophil inflammatory markers in patients undergoing cardiac surgery with long CPB duration. (**a**) There was statistical positive correlation between sCD141 and circulating cfDNA after surgery, at day 1, day 3 day 5 and day 8 respectively. (**b**) There was a negative significant correlation between sCD141 and neutrophil TLR9 at day 1 and day 8 after surgery.

Taken into account that cfDNA might include large amounts of NETs which represent potential inducers of endothelial damage, we further assessed plasma concentrations of soluble thrombomodulin (sCD141) and ICAM-1 which represent common molecular markers of endothelial cell injury (Fig. 3a,b). Whereas sCD141 levels in patients with short-CPB didn’t change over time, sCD141 levels clearly increased after surgery in patients with long CPB duration. Significant intergroup differences were observed immediately after surgery as well as at day 3. Additionally, markedly elevated levels of circulating ICAM-1 were quantified in plasma samples of patients with CPB > 100 min with significant intergroup difference found at day 1 after surgery. In contrast, proinflammatory IL-6 (Fig. 3c) and CRP (Fig. 3d) levels, although tending to be higher in the long-CPB group, did not differ between patients of the two groups. Similarly, no changes in leukocyte counts (Fig. 3e) were found. In support of our hypothesis, cfDNA levels in patients with long CPB duration were significantly positively correlated with sCD141 at all times after surgery despite the limited number of patients included (Fig. 4a). Further on, neutrophil TLR9 expression showed significant negative correlation with sCD141 at day 1 and day 8 after surgery (Fig. 4b), arguing for a protective role of TLR9 on endothelial dysfunction. Importantly, sCD141 and cfDNA did not correlate in patients undergoing surgery with CPB < 100 min (data not shown).

### Circulating cfDNA contributes to patients’ plasma-induced NETosis

**Figure 5:**
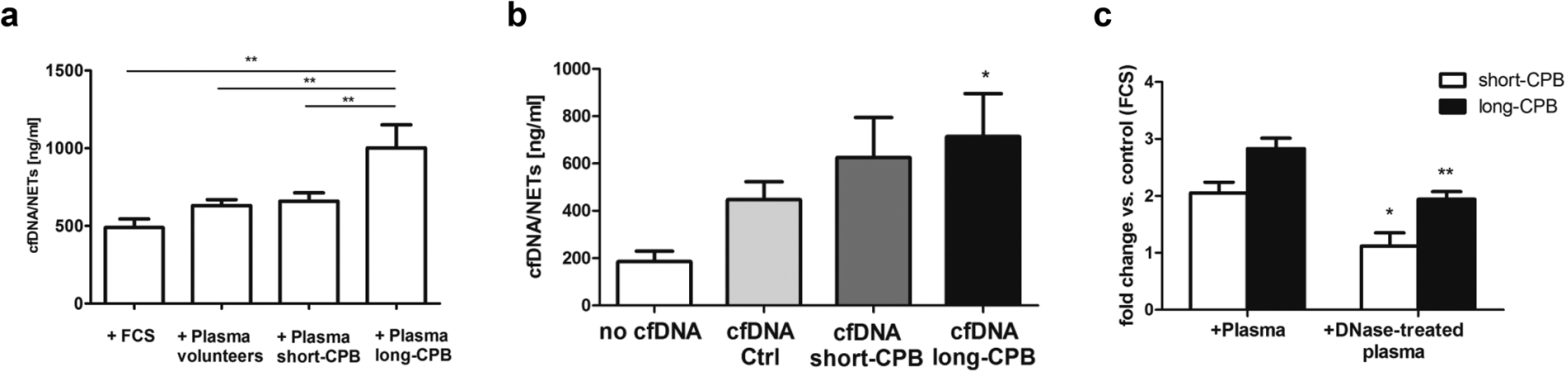
Circulating cfDNA contributes to patient plasma-mediated *in vitro* NETosis. (**a**) Neutrophils from healthy volunteers (1 × 10^6^) were stimulated with 20% pooled plasma from healthy volunteers (n = 3), patients with short-CPB (n = 4) and long-CPB (n = 4), respectively. After 3.5 hours, levels of cfDNA/NETs were quantified in culture supernatants. Data are representative for eight independent experiments. **p < 0.01. (**b**) Circulating cfDNA was isolated from plasma of healthy controls or patients with short (short-CPB) or long (long-CPB) CPB duration, respectively. DNA samples of 4–5 individuals from each group were pooled. Neutrophils from healthy donors (1 × 10^6^) were stimulated with 200 ng DNA for 3.5 hours and cfDNA/NETs were quantified in the culture supernatants (n = 8). *p < 0.05. (**c**) Pooled plasma from patients with short-CPB (n = 4) and long-CPB (n = 4), respectively, was pre-incubated with *DNase I* (190 U/ml) for 2 hours at 37 °C. *DNase I* activity was further inhibited by addition of 5 mM EDTA and neutrophils were incubated in the presence of 20% plasma with or without *DNase I* pretreatment for 3.5 hours. cfDNA/NETs were quantified in the culture supernatants. Fold change vs. control cells cultured in FCS supplemented medium is depicted. Results are representative for four independent experiments *p < 0.05, **p < 0.01.

Inflammatory cytokines are known to play a critical role for NETosis^24^ and circulating mtDNA was found to amplify inflammation^19^ by inducing NETs release via TLR9 activation amongst others^25^. Neutrophils isolated from healthy volunteers, which were already reported to be unstimulated^26^, were activated *in vitro* with pooled plasma from volunteers as well as from cardiac surgery patients (five patients of each group), and cfDNA/NETs were quantified in culture supernatants (Fig. 5a). cfDNA/NETs were significantly increased and highest in supernatants of cells stimulated with plasma from patients with long CPB duration when compared to control groups. To elicit the role of mtDNA for *in vitro* NETs formation, neutrophils were stimulated with DNA isolated from plasma samples of controls and patients (day 1–3 after surgery). By performing Real time PCR the following mean mtDNA contents were detected: 0.0026 in samples from healthy volunteers, 8.53 in plasma from CPB < 100 min and 88.33 in plasma from CPB > 100 min. As shown in Fig. 5b, only stimulation of neutrophils with DNA isolated from plasma of patients with long CPB duration significantly increased cfDNA/NETs levels in culture supernatants. This finding implicates a role of mtDNA for NETosis induction and is consistent with our data showing increase in NETs formation by plasma of patients with long-term CPB. Degradation of plasma cfDNA by *DNase I* markedly impaired the onset of plasma-induced NETosis (Fig. 5c). Nevertheless, cytokines largely contribute to NETs formation, as plasma DNA degradation did not completely inhibit NETosis.

### Patients’ plasma-induced, ROS-independent NETosis does not depend on intracellular TLR9 signaling

**Figure 6:**
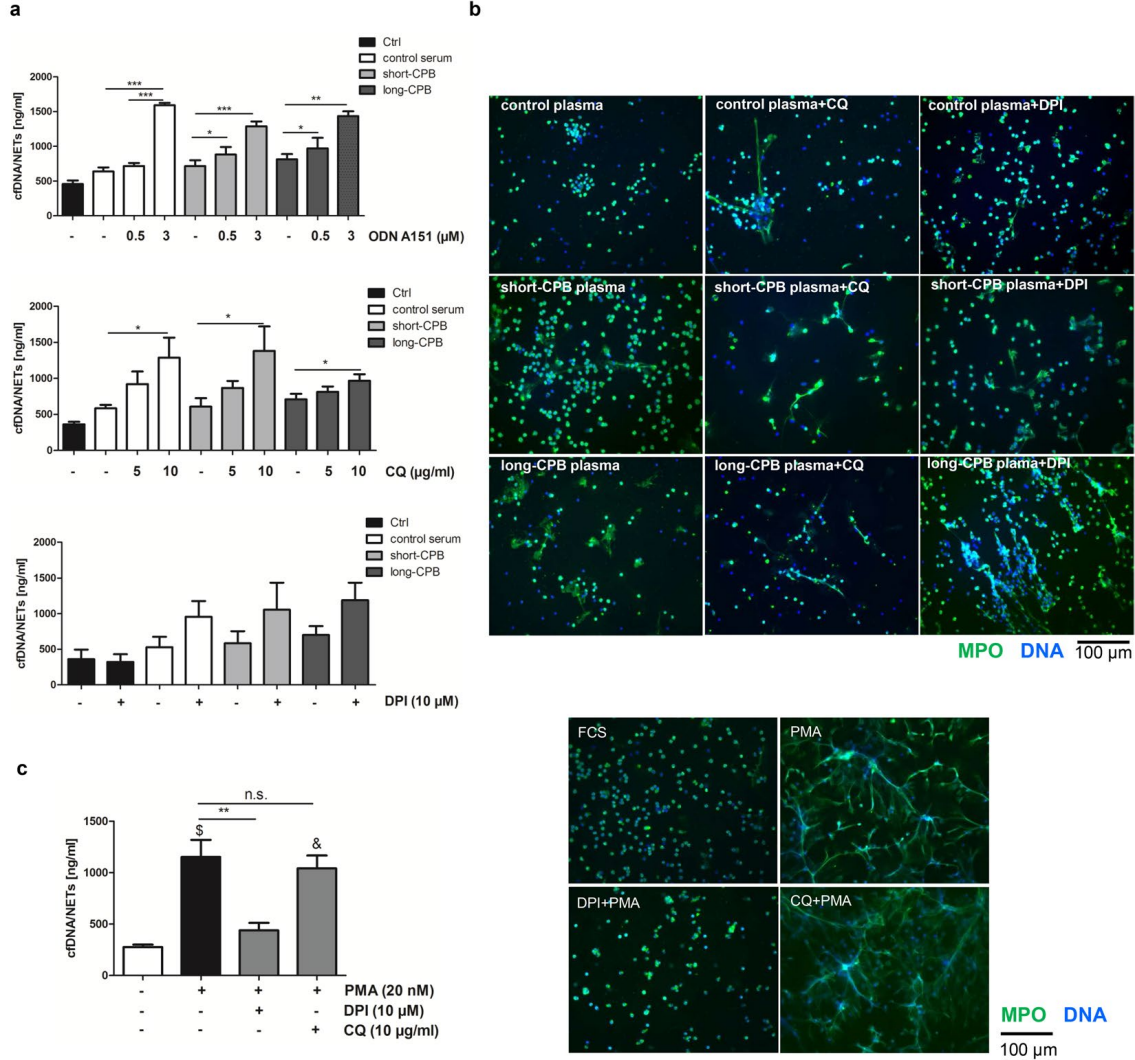
Patient plasma-triggered NETs formation occurs independently of intracellular TLR9 signaling and ROS. (**a**) Neutrophils from healthy volunteers (1 × 10^6^) were pre-cultured with different concentration of ODN A151 (n = 7), chloroquine (CQ) (n = 5) and DPI (n = 5) for 30 min before medium supplementation with 20% pooled plasma from three healthy controls, and four patients with short-CPB or long-CPB, respectively. After incubation for 3.5 hours at 37 °C, cfDNA/NETs were quantified in culture supernatants by Pico green DNA staining. *p < 0.05; **p < 0.01; ***p < 0.001. (**b**) Freshly isolated neutrophils from controls were seeded on poly-D-lysine coated coverslips and allowed to adhere for 30 min in the presence of the inhibitors CQ (10 µg/ml) and DPI (10 µM). Then, cells were stimulated with control plasma (healthy volunteers), pooled plasma of patients with short and long CPB duration for 3.5 h. Cells were stained with anti-MPO antibody (*green*) and couterstained with DAPI (*blue*). Representative images of NETs staining of four independent experiments are depicted. (**c**) In some experiments, neutrophils were stimulated with 20 nM PMA to induce NETosis in the presence or absence of DPI (10 µM) or chloroquine (CQ, 10 µg/ml). DPI completely blocked PMA-induced NET formation. Immunofluorescence data are representative of four independent experiments. **p < 0.01; ^&^p < 0.01, ^$^p < 0.001 vs. control cells without stimulation.

As demonstrated above, plasma cfDNA triggers *in vitro* NETosis in human neutrophils. Therefore, we further examined whether TLR9 is involved in the mechanism of plasma-induced NETs formation. Two common inhibitors of TLR9 signaling, ODN A151 and chloroquine were used for cell culture experiments. Surprisingly, blockage of intracellular TLR9 signaling didn’t suppress plasma-induced NETosis, but rather partially increased NETs release by cultured neutrophils (Fig. 6a,b). Plasma-driven NETosis also did not depend on ROS (Fig. 6a), as in contrast to PMA-triggered NETosis (Fig. 6c), no diminution of NETs formation in the presence of the NADPH inhibitor DPI could be observed. Of note, chloroquine also did not affect PMA-induced NETosis (Fig. 6c).

**Figure 7:**
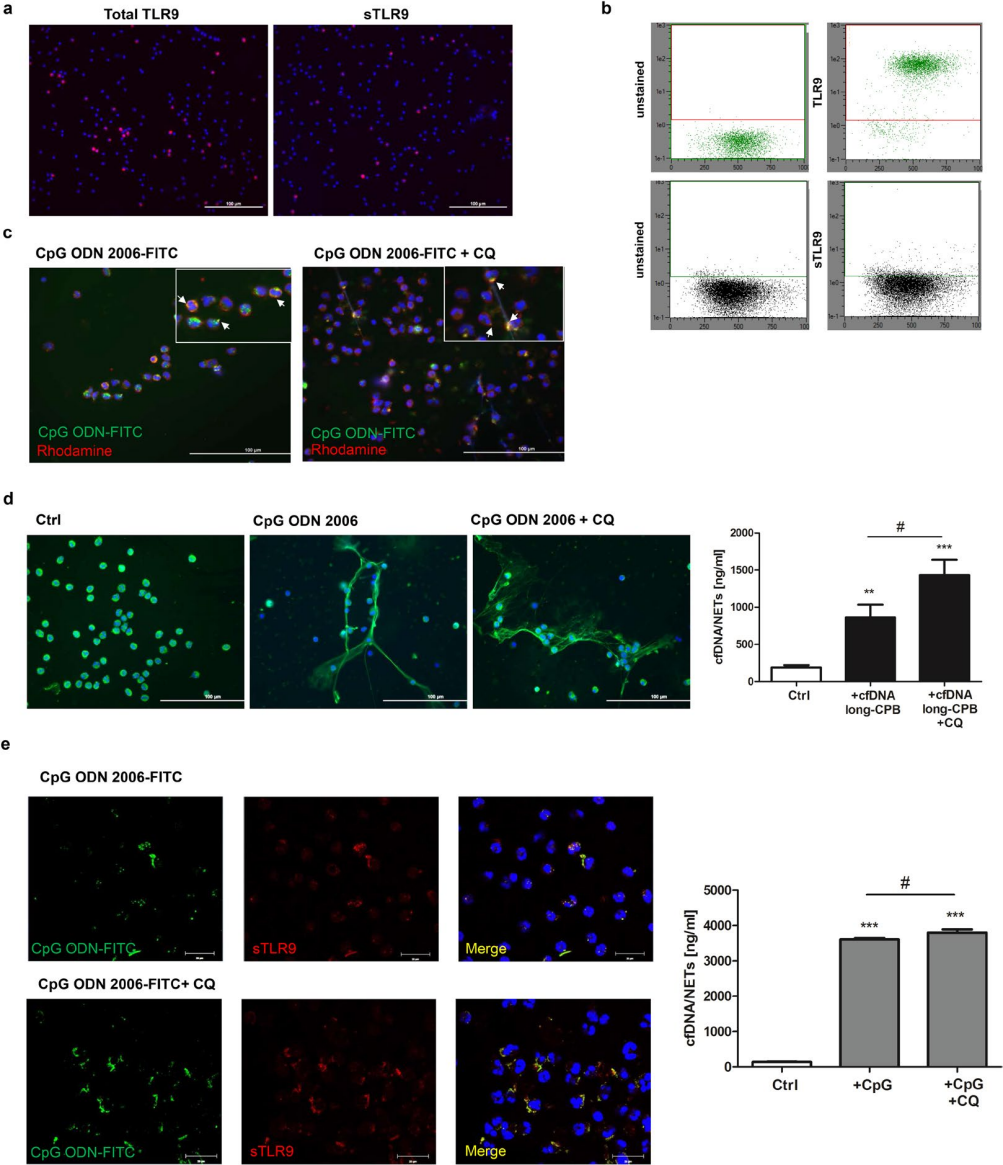
NETosis mediated by sTLR9 occurs independently on intracellular TLR9 signaling. (**a**) Neutrophils were seeded on coated coverslips and cultured for 3 hours. Then, cells were fixed in 4% PFA and permeabilized before incubation with a PE-conjugated anti-TLR9 antibody. To detect sTLR9 expression, cells were left unpermeabilized. Nuclei were counterstained with DAPI and TLR9 expression was visualized by fluorescence microscopy. Results are representative of three independent experiments. (**b**) Neutrophil sTLR9 expression was confirmed by flow cytometry. Briefly, neutrophils (1 × 10^6^) pre-cultured for 3 hours, were fixed, permeabilized and incubated with a PE-conjugated anti-TLR9 antibody. To detect sTLR9, non-permeabilized cells were stained in parallel. TLR9 expression was quantified by MACSQuant Analyzer. Representative dot-blot results are shown (n = 3). (**c**) Neutrophils were preincubated with chloroquine (CQ; 10 µg/ml) for 30 min and then cultured in the presence of 2 µM CpG ODN 2006-FITC for further 3 hours. Plasma membrane staining was achieved by incubation with Rhodamine Wheat Germ Agglutinin. After cell fixation, nuclei were counterstained with DAPI (*blue*) and samples were analyzed by fluorescence microscopy. Colocalization is indicated by white arrowheads. Representative results of four independent experiments are depicted. (**d**) Neutrophils were seeded on coated coverslips, pre-cultured in the presence of chloroquine (10 µg/ml) and further stimulated with 200 ng pooled cfDNA isolated from four patients with CPB > 100 min for 3 hours. NETs were visualized by staining with DAPI (*blue*) and MPO-specific antibody (*green*). Representative images of three independent experiments are depicted. cfDNA/NETs levels were further quantified in culture supernatants after 3.5 hours of stimulation with plasma-dervied cfDNA (n = 7). **p < 0.01; ***p < 0.001 vs. Ctrl; ^#^p < 0.05. (**e**) Neutrophils pre-cultured in the presence of chloroquine or not were stimulated with 2 µM CpG ODN 2006-FITC for 3 hours, fixed and further incubated with a PE-conjugated anti-TLR9 antibody. Colocalization of sTLR9 and CpG ODN was confirmed by confocal microscopy. Data are representative of four independent experiments. For cfDNA/NETs quantification in culture supernatants, neutrophils were incubated with 2 µM CpG ODN 2006 for 3.5 hours (n = 4). ***p < 0.001 vs. Ctrl. ^#^p < 0.05.

Recently, a functional form of the TLR9 receptor on neutrophils’ surface has been reported^27^. We further investigated a possible implication of neutrophil surface TLR9 (sTLR9) for patients’ plasma-triggered NETosis. Neutrophil sTLR9 expression was evidenced by immunofluorescence (Fig. 7a) and flow cytometry (Fig. 7b). In general, most TLR9 was expressed intracellularly. Treatment of neutrophils with the TLR9 agonist CpG ODN 2006-FITC and subsequent staining of the cell membrane with fluorescent wheat germ agglutinin revealed increase of double positive cells, suggesting DNA binding to a surface receptor. However, CpG ODN 2006-FITC was also internalizied by the cells after 3 hours of incubation and chloroquine did not block uptake of oligonucleotides (Fig. 7c). Notably, similar to our findings demonstrating increased plasma-triggered NETosis in the presence of chloroquine, chloroquine significantly increased the amount of NETs in culture supernatants after cell stimulation with plasma-derived cfDNA (Fig. 7d). However, chloroquine alone did not influence NETosis (not shown). As illustrated in Fig. 7e, CpG ODN 2006 showed colocalization with neutrophil sTLR9, whereby only cells displaying high levels of sTLR9 strongly bound to CpG ODN. Hence, CpG ODN 2006, which is similar to bacterial DNA, provoked strong increase in NETs release which was further reinforced by chloroquine.

## Discussion

Despite major improvements in surgical techniques, the use of CPB is still a key requisite helping to bypass circulation out of the heart and lung during the operation. Morbidity associated with CPB can be attributed to the generalized inflammatory response induced by blood-artificial surfaces interactions during extracorporeal circulation and the ischemia/reperfusion implications, including exacerbated inflammatory response resembling SIRS. It is widely recognized that the CPB-induced inflammation is largely mediated by activated neutrophils which shed NETs that have marked effects on inflammation. In this regard, recent evidence has emerged suggesting that NETs might have a role in infectious as well as noninfectious diseases, including, but not limited to, sepsis, SIRS^24,28,29^. In the current study, we demonstrate that circulating cfDNA might represent a feasible early biomarker to assess the onset of CPB-induced inflammation and endothelial damage in patients undergoing open heart surgery with CPB. In addition, we provide evidence for plasma cfDNA-triggered NETosis by a mechanism which is different from the classical TLR9 signaling.

Here, we found that cfDNA levels were significantly increased and highest in patients undergoing cardiac surgery with long-term CPB when compared to those with shorter extracorporeal circulation time. We further speculated that increase in cfDNA might indicate massive neutrophil activation due to prolonged bypass times resulting in excessive NETosis. This assumption was supported by increased creatinine values and increased risk for adverse outcomes found in patients with long CPB duration. However, levels of proinflammatory IL-6, which is also a potent inducer of NETosis^30^, did not differ significantly between groups. On the other hand, proinflammatory biomarkers such as IL-6 and CRP were recently reported to be inappropriate to assess systemic inflammation after cardiac surgery^31^. Importantly, patients with CPB > 100 min displayed significant increase in circulating mtDNA being in line with data reported by other groups^32,33^. As mtDNA did not directly correlate with cfDNA plasma levels, they might not only represent NETs components^17,34^ but rather become continuously released from necrotic tissue. Indeed, NETs were found to damage the host tissue, contribute to the development of autoimmunity and lead to other dysfunctional outcomes, including metastasis, thrombosis and inappropriate coagulation^29^. The detrimental effects of long-term CPB and NETs formation are supported by our observation showing a positive correlation between cfDNA and sCD141 in patients’ plasma starting immediately after surgery and persisting until at least day 8. However, due to technical and logistic issues, a very small number of patients were included in the mentioned anlayses representing a major limitation of this study. Hence, although our data strongly suggest a link between cfDNA and endothelial damage, further analyses including a larger number of patients or an established animal model are necessary to confirm our findings.

Furtheron, if mtDNA, which was also elevated in patients with CPB > 100 min, directly contributes to endothelial damage or is rather a consequence of it cannot be definitely assessed. Activation of the immune system by mtDNA is based on the recognition and binding of unmethylated CpG dinucleotides to endosomal TLR9. In recent years, neutrophils have been found to express sTLR9 which is potentially able to engage DAMPs, such as mtDNA released from necrotic cells during sterile inflammation. Till date, only a few studies exist describing the function of neutrophil sTLR9^27,35^ and they largely propose an involvement in detrimental effects by provoking massive proinflammatory cytokine production. Thus, sTLR9-expressing neutrophils are key players in inducing rapid inflammation^36^, promoting sepsis or SIRS^37^. Of interest, we found that plasma-triggered NETosis does not involve intracellular TLR9 signaling. Treatment of cells with inhibitors of TLR9 signaling, e.g. chloroquine, strongly increased plasma-mediated NETosis, suggesting that blockage of intracellular TLR9 signaling might strengthen signaling mediated by sTLR9. Indeed, we demonstrate here for the first time that neutrophils expressing sTLR9 bind high amounts of extracellular DNA, yielding in NETosis, again pointing for a role of sTLR9 as a mediator of rapid immune responses. sTLR9 and NETosis, respectively, were not suppressed by the inhibitors used in this study. Thus, our results are in line with the findings reported by Lindau *et al*. demonstrating that the signaling pathway mediated by sTLR9 is different from the classical TLR9 pathway^27^. As NETs are known to stimulate TLR9 in dendritic cells^38^, we suggest that cfDNA, enclosing NETs, might amplify the inflammatory response by activating sTLR9 and NETosis in adjacent neutrophils, whereby a possible involvement of other DNA sensors is not negligible. Mechanistically, cfDNA-triggered NETosis is independent on ROS production. In this regard, ROS-independent NETosis has already been reported as a very rapid process stimulated by *Leishmania amazonensis* ^39^ amongst others. Additionally, patient plasma-triggered NETosis does not solely depend on cfDNA and other proinflammatory cytokines and DAMPs are also implicated in this process.

It has been found that mtDNA upregulates TLR9 expression in macrophages and also neutrophils^23,27^. Similarly, plasma from patients with acetaminophen-induced acute liver failure upregulated neutrophil TLR9 expression *in vitro* and this increase was abrogated by preincubation with *DNase I* ^40^. Here, neither an *in vivo* correlation between TLR9 and mtDNA nor an *in vitro* receptor upregulation by patients’ plasma or plasma cfDNA could be detected (unpublished results). Although patients with CPB > 100 min displayed rapid TLR9 upregulation after surgery, it has to be taken into account that we did not differentiate between endosomal and sTLR9 expression representing a further major limitation or our study. Thus, *in vivo* sTLR9 regulation, especially after prolonged bypass, cannot be surely excluded. On the other hand, negative correlation of total TLR9 and sCD141 might indicate TLR9-linked endothelial protection. On the basis of our data, we speculate that increased NETosis in patients with prolonged CPB duration is rather due to an upregulation of neutrophil sTLR9 whereby only a minor implication of endosomal TLR9 can be assumed.

In summary, cfDNA represents an early biomarker for CPB-induced inflammation and a potential mediator of endothelial damage after cardiac surgery with prolonged bypass duration. To our knowledge, our study demonstrates for the first time that cfDNA boosts inflammation, amplifying NETosis by a novel mechanism which is independent on endosomal TLR9 and ROS and at least partially depends on sTLR9. However, the therapeutic value of cfDNA-targeting molecules/enzymes and the functional role of neutrophil sTLR9 in inflammation should be validated in future clinical and experimental studies using animal CPB models.

## Methods

### Patients and sample collection

This prospective pilot study was approved by the Ethics Committee of the Medical Faculty of the University of Cologne (#15–393). Written informed consent was obtained from all participants at the time of admission. Forty-eight patients undergoing any kind of on-pump cardiac surgery between April 2016 and March 2017, aged 18 years or older were enrolled in this study. Exclusion criteria were withdrawal of patients’ consent, infectious diseases and malignancy. In addition, patients with known preexisting acute or chronic immunological disorders or systemic immunosuppression were excluded from the study. The preoperative risk for operative mortality was evaluated by means of the additive and logistic EuroSCORE^41^. Additionally, the ICU scores SAPS II and TISS^42,43^ were calculated for each patient. Included patients were stratified according to the time on CPB (cut-off: 100 min)^44^. In addition, fifteen patients undergoing off-pump coronary artery bypass (OPCAB) surgery were included (#13–230).

Fourteen healthy volunteer blood donors (4 male, 10 female) aged between 21 and 60 years served as controls. Exclusion criteria were age under 18 years, recent or ongoing infection, cardiovascular disorders, immunosuppressive therapy or pregnancy.

Blood samples were collected into tubes containing lithium heparin (Sarstedt, Cologne, Germany) at the day of admission, immediately after surgery as well as postoperatively at day 1, day 3, day 5 and day 8. In some cases, no blood samples were collected from patients or patients were released from the hospital before day 8 after surgery, respectively. Blood samples were immediately used for neutrophil isolation and subsequent analyses. Plasma samples were harvested by centrifugation (10 min at 3000 × *g*) and stored at −80 °C until further processing.

### Isolation of human neutrophils, culture conditions and cell activation

Human neutrophils were separated from whole blood by discontinuous density-gradient centrifugation on Percoll (Millipore)^45^. In brief, blood was carefully layered onto the percoll gradient and centrifuged at 2000 rpm for 30 min without break. After removal of PBMCs, erythrocytes were lysed using isotonic ammonium chloride solution (155 mM NH_4_Cl, 10 mM KHCO_3_, 0.1 mM EDTA) at 4 °C for 10 min. The purity and viability of neutrophils were >95% as examined by flow cytometry analysis and trypan blue exclusion, respectively.

For NETs induction, human neutrophils (2.5 × 10^6^/ml) were pre-cultured with chloroquine (5 µg/ml; 10 µg/ml, Cayman Chemical), DPI (10 µM, Cayman Chemical), or ODN TTAGGG (A151; 0.5 µM; 3 µM, Invivogen) in medium supplemented with 2% FCS for 30 min. Then, 20% of pooled plasma from patients, volunteers, or FCS was added. After 3.5 hours, cell culture supernatants were harvested and stored at −80 °C.

### Determination of TLR9 expression by flow cytometry

To assess TLR9 protein expression in neutrophils, 1 × 10^6^ neutrophils were fixed in 4% paraformaldehyde (PFA), permeabilized in methanol and stained with PE-labelled rat anti-human TLR9 antibody (clone eB72-1665, BD Pharmingen) in 0.1% Triton X-100 and 3% FCS in cell wash buffer (BD) for 30 min in the dark. After washing two times with 0.1% Triton X-100 and 3% FCS in cell wash buffer (BD), cells were analyzed by flow cytometry using a MACSQuant Analyzer (Miltenyi Biotec) or FACSCalibur (BD). For this, neutrophils were gated using forward and side scatter. For surface TLR9 staining, non-permeabilized cells were incubated with the same antibody in cell wash buffer with 3% FCS for 30 min on ice. Cells were washed twice and immediately analyzed by flow cytometry.

### Quantification of cfDNA and NETs

Plasma levels of cfDNA were quantified by Quant-iT Pico Green dsDNA assay by following the manufacturer’s instructions (Invitrogen GmbH, Darmstadt, Germany)^46^. For the quantification of NETs in cultured neutrophils, cells were suspended and centrifuged at 300 × g for 5 min at 4 °C. Supernatants containing NETs were collected and quantified by Pico Green staining. The fluorescence intensity reflects the amounts of DNA and was measured at excitation and emission wavelengths of 485 nm and 530 nm, respectively in a microplate reader (Victor3, PerkinElmer). A standard calibration curve by means of defined calf thymus DNA (Sigma Aldrich) amounts ranging from 0 to 2 µg/ml has been used in all analyses.

### Measurement of plasma *DNaseI* activity

*DNase I* activity in patients’ plasma was measured by the method previously described with some modifications^47^. Briefly, plasma samples were diluted 1:10 with PBS + 10 mM MgSO_4_ supplemented with 1 µg/ml of calf thymus DNA (Sigma-Aldrich) and stained with Pico Green (Invitrogen). To allow digestion of DNA by plasma *DNase I*, samples were incubated for 2 h at 37 °C. The intensity of Pico Green fluorescence was measured at time points 0 h and 2 h using a microplate reader (Victor3, Perkin Elmer). Relative *DNase I* activity was expressed as the ratio of relative fluorescence units measured at time point 0 h and 2 h, whereby high values may reflect high *DNase I* activity.

### Quantification of IL-6, soluble thrombomodulin and ICAM-1 by ELISA

Plasma IL-6 and ICAM-1 were determined by using the Human IL-6 DuoSet Elisa (R&D Systems, Wiesbaden-Nordenstadt, Germany) and the Human ICAM-1/CD54 DuoSet Elisa (R&D Systems, Wiesbaden-Nordenstadt, Germany) according to the manufacturer’s instructions. For the quantification of soluble thrombomodulin (sCD141) levels, the CD141/Thrombomodulin Elisa (Diaclone, Besancon Cedex, France) has been used.

### DNA isolation from plasma

200 µl of plasma in a total volume of 500 µl were mixed with 5 µl Triton X-100 and heat denaturated at 98 °C for 5 min. Samples were placed on ice for 5 min and DNA was extracted with an equal volume of phenol chloroform isoamyl alcohol (25:24:1, Sigma Aldrich) and centrifuged for 10 min at 4 °C. The aqueous phase was precipitated overnight with 1/10 volume of 3 M NaOAc and 2.5 volume 100% ethanol at −20 °C. The DNA pellet was washed twice with ethanol, air-dried and resuspended in 20–30 µl of ddH_2_O.

### Heparinase I treatment of samples

Digestion of plasma samples using heparinase I (Sigma Aldrich) was performed as previously described^48^. In brief, heparinase I (50 U) was dissolved in 50 mM Tris (pH 7.5) and 1 mM CaCl_2_, and stored at −20 °C. 5 µl of freshly isolated DNA was added to 19 µl of heparinase I and samples were incubated for 2 hours at 25 °C. 5 µl of the treated DNA solution was used as template in subsequent real time PCR reactions.

### Quantification of mitochondrial and nuclear DNA in plasma samples by Real time PCR

Relative mitochondrial DNA (mtDNA) copy numbers were quantified by Real time PCR and normalized by simultaneous detection of nuclear DNA (nucDNA). The ABI StepOne Plus Real-Time PCR System (Applied Biosystems) was used to amplify GAPDH, the nuclear gene, and the mtDNA-encoded ATPase 8 gene by using previously published primer sequences^49^. PCR was performed in 12.5 µl of total reaction volume containing 5 µl treated DNA (10 ng-20 ng), 6.25 µl 2 × SYBR Green PCR master mix (Applied Biosystems) and 800 nM forward and reverse primers. The following thermal cycling conditions were used: denaturation step at 95 °C for 10 min, 40 cycles of 60 sec at 60 °C and 15 sec at 95 °C. All samples were analyzed in duplicate. The average threshold cycle number (Ct) values were determined in the same quantitative PCR run. The threshold was set in the same level for each run. The level of mtDNA was calculated relative to GAPDH as followed: relative copy number 2^∆Ct^, whereby ∆Ct = Ct_nuc_ − Ct_mit_.

### Immunofluorescence staining and fluorescence microscopy

For NETs staining, freshly isolated neutrophils (1 × 10^6^) were seeded on poly-D-lysine coated coverslips and incubated in the presence of 10 µM DPI or 10 µg/ml chloroquine for 30 min. Then, cells were stimulated with serum or phorbol 12-myristate 13-acetate (PMA, Sigma Aldrich) for 3.5 hours. After that, samples were fixed with 4% PFA, blocked and incubated with a polyclonal rabbit anti-myeloperoxidase antibody (ab9535, 1:100, Abcam) and a secondary goat anti-rabbit Alexa Fluor 488-conjugated antibody (1:1000; Cell Signaling Technology). To demonstrate binding of DNA to neutrophils’ surface, PMN were incubated with FITC-conjugated CpG ODN 2006 (2 µM, Invivogen). Plasma membrane was stained with Rhodamine Wheat Germ Agglutinin (5 µg/ml, Vector Laboratories). For colocalization studies of DNA and surface TLR9, neutrophils were fixed and incubated with PE-conjugated anti-TLR9 antibody (clone eB72-1665, Invitrogen). For intracellular TLR9 staining, cells were permeabilized with PBS + 1% FCS + 0.3% TritonX-100 prior incubation with anti-TLR9 antibody. Isotype control was used as control. All cells were counterstained with DAPI (Cayman Chemical) and specimens were mounted in Dako fluorescent mounting medium (Dako). Cells were finally visualized by immunofluorescence microscopy (Eclipse Ti-U 100, Nikon) using the NIS Elements BR 3.10 software package. Neutrophil sTLR9 surface expression and colocalization with FITC-CpG-ODN 2006 were confirmed by confocal microscopy using the Zeiss LSM 710 Inverted Meta confocal microscope (Carl Zeiss).

### Statistical analyses

Data were analyzed with GraphPad Prism 5 software. Patient data are presented as box plots representing the median (heavy line in boxes) and the 25^th^ and 75^th^ percentiles. Whiskers indicate the minimum and maximum values, respectively. Experimental data are presented as means with standard error of the mean (SEM). The difference among non-normally distributed data was determined by the Kruskal-Wallis approach with Dunn’s post hoc. Unpaired data of two groups were analyzed using the Mann-Whitney test. Spearman correlation coefficient (*r*) was used to determine the presence or absence of a correlation between variables. Normally distributed unpaired data of multiple groups were analyzed with ANOVA and Newman Keuls post-hoc test. P-value less than 0.05 was considered as statistically significant.

### Data availability statement

All data generated or analyzed in this study are available from the corresponding author on reasonable request.

All experiments were performed in accordance with relevant guidelines and regulations.

## Electronic supplementary material

Supplementary Information
