# Supplementary material for: cfDNA correlates with endothelial damage after cardiac surgery with prolonged cardiopulmonary bypass and amplifies NETosis in an intracellular TLR9-independent manner
Source: Sci Rep. 2017 Dec 12;7:17421. doi: 10.1038/s41598-017-17561-1 (PMC5727170; doi:10.1038/s41598-017-17561-1)
Supplement: Supplementary file 1 — Supplementary Information [file 41598_2017_17561_MOESM1_ESM.doc]

**cfDNA correlates with endothelial damage after cardiac surgery with prolonged cardiopulmonary bypass and amplifies NETosis in an intracellular TLR9-independent manner**

Adnana Paunel-Görgülü^1*^, Max Wacker^1^, Mouhamed El Aita^1^, Shoreshfan Hassan^1^, Georg Schlachtenberger^1^, Antje Deppe^1^, Yeong-Hoon Choi^1^, Elmar Kuhn^1^, Thorsten O. Mehler^2^, Thorsten Wahlers^1^

^1^Department of Cardiothoracic Surgery, Heart Center of the University of Cologne, Cologne, Germany

^2^Department of Anaesthesiology and Intensive Care Medicine, University Hospital Cologne, Cologne, Germany

**Supplementary Table S1.** Baseline characteristics of patients undergoing off-pump surgery

|  |  |
| --- | --- |
| N | 15 |
| Age (years) | 72.79±19.95 |
| Female (%) | 20 |
| BMI | 27.24±1.94 |
| Operation time (min) | 171.21±27.98 |
| ICU stay (d) | 2.5±2.8 |
| Hospital stay (d) | 16.71±4.82 |
| Ventilation time (h) | 16.41±6.2 |
| Euroscore additive | 5.6±1.89 |
| Euroscore logistic | 6.2±5.3 |
| SAPS II | 28.7±9.1 |
| TISS | 27.9±3.9 |
| Risk factors |  |
| Diabetes mellitus | 1(6.67%) |
| COPD | 3(20.00%) |
| Hypertension | 15(100%) |
|  |  |

Data are presented as mean±SD.
